# Multifaceted benefits of xylitol in oral health: from caries prevention to periodontal therapy

**DOI:** 10.6026/973206300220588

**Published:** 2026-01-31

**Authors:** Nidhiswi Yeruva, Luisana Rodriguez, Melania Romero, Anjali Aryal, Leonardo Acosta Pina, Mihriban Saygi

**Affiliations:** 1 Department of Dental Surgery, Kamineni Institute of Dental Sciences, Nalgonda, India; 2 Department of Dental Surgery, School of Dentistry, University of Carabobo, Naguanagua-Venezuela; 3 Department of Dental Surgery, School of Dentistry, University of Zulia, Maracaibo-Venezuela; 4 Department of Dental Surgery, City Dental College, University of Dhaka, Dhaka-Bangladesh; 5 Department of Dental Surgery, School of Dentistry, University Santa Maria, Caracas-Venezuela; 6 Department of Dental Surgery, Necmettin Erbakan University Faculty of Dentistry, Konya/ Turkey

**Keywords:** Xylitol, dental caries prevention, periodontal therapy, xerostomia, oral health promotion

## Abstract

Xylitol, a naturally occurring sugar alcohol, exhibits multifaceted benefits in dentistry, including inhibition of cariogenic bacteria, suppression of biofilm formation and stimulation of salivary flow. Therefore, it is of interest to review synthesizes current evidence on its role in caries prevention, periodontal therapy and management of xerostomia, candidiasis and halitosis. Clinical and public health implications have been discussed, with emphasis on optimal dosage, delivery methods and integration into preventive strategies. Emerging evidence highlights its relevance as a safe and adjunctive modality within evidence-based preventive and therapeutic protocols. Future studies should include rigorously designed randomized controlled trials and translational studies to establish standardized guidelines for integrating it into clinical practice and community-based oral health programs.

## Background:

Xylitol is a naturally occurring sugar alcohol widely recognized for its beneficial effects on oral health, particularly in the prevention of dental caries [1]. Its effectiveness is supported by both its unique chemical properties and its ability to interfere with the cariogenic process in multiple ways [2]. One of the most important actions of xylitol is its ability to reduce the levels of *Streptococcus mutans*, the primary bacterium responsible for tooth decay [3]. Unlike fermentable sugars such as sucrose or glucose, xylitol cannot be metabolized by *S. mutans*. When these bacteria take up xylitol, their energy production is disrupted and their growth is inhibited. This leads to a decrease in bacterial adhesion to the tooth surface and a reduction in acid production, both of which are critical factors in the formation of dental plaque and the initiation of carious lesions [4]. Another key benefit of xylitol is its capacity to stimulate saliva flow, especially when consumed in the form of chewing gum. Increased salivary flow enhances several protective functions in the oral cavity. It helps neutralize acids produced by bacterial metabolism, facilitates the remineralization of demineralized enamel and aids in clearing food debris and bacteria from the mouth [5]. This protective effect is particularly valuable for individuals with reduced salivary flow, such as patients with xerostomia (dry mouth), who are at higher risk of developing dental caries [6]. Xylitol also contributes to the control of dental plaque. It reduces the stickiness of plaque, which makes it less likely to accumulate on tooth surfaces. This is important because plaque buildup creates a localized acidic environment that promotes enamel demineralization and decay [7]. Furthermore, xylitol indirectly improves enamel strength by supporting the remineralization of early enamel lesions. When consumed regularly, especially after meals, xylitol promotes conditions that favor mineral deposition in demineralized enamel, thus helping to reverse the early stages of tooth decay [8]. Several clinical studies have confirmed that regular xylitol use leads to a significant reduction in the incidence of dental caries. These benefits are especially notable in children at high risk of developing cavities and in individuals with compromised saliva production [9]. For xylitol to be effective in caries prevention, a daily intake of 5 to 10 grams is recommended, divided into at least three exposures throughout the day. This is usually achieved by chewing xylitol-containing gum or consuming xylitol-sweetened candies for 5 to 10 minutes after meals [10], Xylitol is available in various dental care products, including chewing gum, lozenges, syrups for young children, toothpaste and mouth rinses. Its safety, pleasant taste and preventive benefits make it a valuable addition to daily oral hygiene practices aimed at reducing dental caries [11]. Therefore, it is of interest to report the relevance of xylitol application in dentistry.

## Xylitol in caries prevention:

## Mechanisms and considerations:

Xylitol is a naturally occurring five-carbon sugar alcohol that has demonstrated significant anticariogenic properties. Unlike fermentable sugars, xylitol cannot be metabolized by *Streptococcus mutans*, leading to reduced acid production and a stable oral pH. This helps prevent enamel demineralization and dental caries [12]. Its primary mechanism involves a metabolic dead-end process: *S. mutans* mistakenly take up xylitol, converting it to xylitol-5- phosphate-a compound the bacteria cannot process, thereby depleting energy and suppressing bacterial growth [13]. Additionally, xylitol impairs biofilm formation by reducing extracellular polysaccharide production and shifts the oral flora toward less acidogenic species [14]. Xylitol also stimulates salivary flow when used in gum form, enhancing mechanical cleansing, acid neutralization and enamel remineralization [5]. These combined actions make xylitol particularly beneficial for children, elderly patients and those with xerostomia or limited access to dental care [9]. However, its benefits depend on consistent use and appropriate dosing, which can be challenging for some patients. Excessive intake may cause gastrointestinal discomfort, such as bloating or diarrhea [15, 16]. Additionally, xylitol is extremely toxic to dogs, leading to liver failure and hypoglycemia [17]. Future studies should emphasize long-term clinical trials, ideal dosage schemes and improved delivery systems to enhance compliance and clinical outcomes [18].

## Periodontal therapy:

Xylitol, while initially marketed as a sugar substitute, has gradually gained recognition for its positive effects on oral health, particularly in the prevention of dental caries [19]. Its resistance to fermentation by oral bacteria enables xylitol to interfere with acid production by cariogenic and periodontal pathogens, thereby reducing the risk of demineralization and disease progression [20, 21]. Periodontitis is a destructive inflammatory disease that affects the supporting structures of the teeth. It results from host responses to bacterial endotoxins in localized plaque and leads to the destruction of connective tissue and alveolar bone, ultimately causing tooth loss if left untreated [22]. Two major bacterial species implicated in periodontitis are Porphyromonas gingivalis and Aggregatibacter actinomycetemcomitans. These pathogens induce the expression of pro-inflammatory cytokines, contributing to the degradation of the periodontium and bone resorption [23]. Recent investigations into xylitol have explored its anti-inflammatory effects, especially its impact on the immune response to periodontal pathogens. Preliminary evidence suggests that xylitol reduces the virulence of *P. gingivalis* by lowering cytokine expression and blocking lipopolysaccharide production, which lessens the localized inflammatory response [20, 21]. Additionally, xylitol stimulates salivary flow, facilitating mechanical removal of bacteria and buffering acidic metabolites. Saliva contains antimicrobial peptides and enzymes that enhance mucosal immunity, so xylitol's sialogogue effect is especially significant for patients with xerostomia or reduced salivary function [24, 25]. For such patients, xylitol-containing products offer added therapeutic benefits. Clinicians have integrated xylitol into preventive care protocols for patients with early gingivitis or active periodontitis. It is commonly available in the form of chewing gums, lozenges, mouthwashes, or toothpastes, delivery methods that allow for localized application. In addition to its antimicrobial properties, xylitol is biocompatible and suitable for extended use without known adverse effects [11]. It can be easily incorporated into conventional oral hygiene routines, making it a practical adjunct to traditional therapies. While current data support the potential of xylitol in periodontal therapy, future studies are needed to determine optimal dosage, duration and possible synergistic effects with other agents. Clinical trials will be essential to establish its long-term efficacy and safety across diverse patient populations. In summary, xylitol has evolved from a mere sugar substitute to a multifunctional compound of great interest in dental medicine [23]. Its ability to inhibit pathogenic bacteria, modulate immune responses and promote salivary function represents a promising strategy for maintaining both periodontal and general oral health [3].

## Xerostomia, Candidiasis and Halitosis:

Research on dental health has focused more on xylitol, a naturally occurring five-carbon sugar alcohol, because of its saliva- stimulating, antibacterial and non-cariogenic qualities. Xylitol inhibits demineralization because, in contrast to fermentable carbohydrates, it is not broken down by oral bacteria to create acids. Its incorporation into treatment plans has proven advantageous in the treatment of three oral diseases that substantially lower quality of life: xerostomia, candidiasis and halitosis [26].

## Xerostomia:

Dry mouth, or xerostomia, is frequently caused by radiation therapy, polypharmacy and autoimmune conditions, including Sjögren syndrome. Mucosal infections, higher caries risk and difficulty masticating can result from reduced salivary flow. By stimulating the gustatory and mechanical senses, xylitol, when added to chewing gum or lozenges, increases salivary flow. Improved mucosal lubrication, pH buffering and oral hydration are all facilitated by increased saliva [18]. Chewing xylitol gum two to three times a day has been demonstrated in clinical research to significantly reduce the symptoms of dry mouth and prevent plaque formation [24].

## Oral candidiasis:

Candida albicans is the primary cause of oral candidiasis, a fungal infection that is particularly prevalent in immunocompromised individuals, those with xerostomia and those who wear dentures. By interfering with the metabolic processes and biofilm formation of Candida species, xylitol has antifungal action [27]. By stimulating saliva, this sugar alcohol promotes spontaneous clearance and prevents fungi from adhering to mucosal surfaces. Its application has been demonstrated to lower infection rates and severity, especially in susceptible groups [28].

## Halitosis:

In periodontal pockets and on the dorsum of the tongue, anaerobic bacteria such as Porphyromonas gingivalis and Prevotella intermedia produce volatile sulfur compounds (VSCs), including hydrogen sulfide and methyl mercaptan, which cause halitosis. In order to lower oral pH and dilute VSCs, xylitol decreases bacterial colonization and increases salivary flow [29]. It has been demonstrated that regular use of gum or mints containing xylitol reduces oral malodor, particularly when combined with good oral hygiene habits [30]. Apart from its well-established function in preventing dental cavities, xylitol has a variety of therapeutic and preventive uses in the treatment of halitosis, xerostomia and oral candidiasis. Its ability to increase mucosal protection, control microbial biofilm dynamics and stimulate salivary flow highlights its value as a community-level health strategy, in addition to individual treatment [27]. In keeping with the principles of preventive dentistry, xylitol is a non-fermentable, biocompatible sugar alternative that has antibacterial and immunomodulatory properties [15]. Its low-risk, easily accessible intervention with an impact on individuals and populations is further established by its incorporation into standard oral care products and public health initiatives, such as school- based nutrition programs, dietary guidelines and geriatric oral hygiene protocols [29].

## Public Health and Maternal/Community Use:

One of xylitol's additional benefits is its broad systemic effects. It inhibits potential skin pathogens and remains undigested in the gastrointestinal tract, where it undergoes fermentation. Certain species within the genus Anaerostipes have been reported to ferment xylitol, resulting in butyrate production [7]. In contrast, common strains of *Lactobacillus* and Bifidobacterium appear unable to utilize xylitol as a growth substrate, thereby alleviating constipation and contributing to increased bone density. In addition to its various physiological effects, xylitol plays a regulatory role in the immune system, helping to lower the incidence of respiratory tract infections and otitis media. Due to its low caloric content, it may also support a healthy body weight. While several health benefits of xylitol have been identified, others remain insufficiently studied [31]. One of xylitol's most notable preventive effects is its role in minimizing the mother-to-child transmission of *mutans streptococci* (MS), a key contributor to early childhood caries. A longitudinal study from Finland found that mothers who consistently used xylitol chewing gum had significantly lower MS levels in their saliva. Consequently, their children showed delayed bacterial colonization and a reduced incidence of caries by age five [32]. Comparable findings were observed in a Japanese study, where prenatal and postpartum xylitol use correlated with a noticeable decline in early MS colonization in infants [33, 10]. A national comparison of adolescent health behavior surveys over 14 years demonstrated a remarkable increase in xylitol chewing gum use among 12- to 18-year-olds. Interestingly, socioeconomic background and urbanization level did not significantly influence xylitol use. This shift in consumption patterns likely resulted from comprehensive public health initiatives in Finland, including educational campaigns and industry collaboration [32]. Policy-wise, integrating xylitol-containing products into national oral health strategies has the potential to reduce the long-term financial burden of dental care. Some Nordic nations have already acknowledged the extended benefits of xylitol and incorporated it into their official dental health policies [17]. However, cost-related barriers and limited public awareness still present challenges to widespread implementation [33]. In summary, xylitol's role in dentistry extends well beyond the prevention of dental decay. Its contributions to microbial control, salivary stimulation and public health integration make it a valuable and patient-friendly element of modern preventive strategies [13].

## Limitations and future perspectives:

In spite of the fact that xylitol can have a profound effect in dental caries prevention, a few limitations are worth mentioning. It shows a dose-dependent anticariogenic effect; conditions of regular and adequate consumption are required to ensure the therapeutic effect is maintained [34]. This property has the potential to adversely impact patient adherence, especially in populations where the use of chewing gum or lozenges may not be culturally appropriate or convenient. Also, patients may not take xylitol-containing products with awareness of correct timing or simply due to a lack of motivation, forgetfulness, or misunderstanding of its preventive value. Additionally, the variability in individual response and the possibility of gastrointestinal discomfort at higher doses may further limit its widespread use [19]. Future perspectives should focus on improving delivery systems that enhance compliance and acceptance, such as incorporation into commonly used dental products (*e.g.*, toothpaste, mouth rinses) and food items. Additionally, there is a need for more robust, long-term clinical trials across diverse populations to establish standardized dosing guidelines and evaluate synergistic effects with other preventive agents. Expanding public health initiatives and educational programs to raise awareness about xylitol's benefits may further support its integration into everyday oral care and community health strategies [34].

## Conclusion:

Xylitol plays a key role in oral health by effectively preventing dental caries, promoting enamel remineralization and limiting plaque formation. Beyond caries prevention, it offers additional benefits in managing xerostomia, oral candidiasis, halitosis and periodontal diseases, though excessive consumption may cause gastrointestinal discomfort in some individuals. It is a cost-effective and accessible adjunct in preventive dentistry, with the potential to improve oral health outcomes globally when used appropriately as part of comprehensive oral care routines, particularly in reducing caries incidence among high-risk and vulnerable populations.
